# Anti-Cancer Properties of Theaflavins

**DOI:** 10.3390/molecules26040987

**Published:** 2021-02-13

**Authors:** Eric J. O’Neill, Deborah Termini, Alexandria Albano, Evangelia Tsiani

**Affiliations:** 1 Faculty of Applied Health Sciences, Brock University, St. Catharines, ON L2S 3A1, Canada; eo15nv@brocku.ca (E.J.O.); dt14fl@brocku.ca (D.T.); aa13ef@brocku.ca (A.A.); 2 Centre for Bone and Muscle Health, Brock University, St. Catharines, ON L2S 3A1, Canada

**Keywords:** cancer, nutraceuticals, theaflavins, polyphenols, apoptosis, black tea

## Abstract

Cancer is a disease characterized by aberrant proliferative and apoptotic signaling pathways, leading to uncontrolled proliferation of cancer cells combined with enhanced survival and evasion of cell death. Current treatment strategies are sometimes ineffective in eradicating more aggressive, metastatic forms of cancer, indicating the need to develop novel therapeutics targeting signaling pathways which are essential for cancer progression. Historically, plant-derived compounds have been utilized in the production of pharmaceuticals and chemotherapeutic compounds for the treatment of cancer, including paclitaxel and docetaxel. Theaflavins, phenolic components present in black tea, have demonstrated anti-cancer potential in cell cultures in vitro and in animal studies in vivo. Theaflavins have been shown to inhibit proliferation, survival, and migration of many cancer cellswhile promoting apoptosis. Treatment with theaflavins has been associated with increased levels of cleaved poly (ADP-ribose) polymerase (PARP) and cleaved caspases-3, -7, -8, and -9, all markers of apoptosis, and increased expression of the proapoptotic marker Bcl-2-associated X protein (Bax) and concomitant reduction in the antiapoptotic marker B-cell lymphoma 2 (Bcl-2). Additionally, theaflavin treatment reduced phosphorylated Akt, phosphorylated mechanistic target of rapamycin (mTOR), phosphatidylinositol 3-kinase (PI3K), and c-Myc levels with increased expression of the tumour suppressor p53. This review summarizes the current in vitro and in vivo evidence available investigating the anti-cancer effects of theaflavins across various cancer cell lines and animal models.

## 1. Introduction

### 1.1. Cancer

Cancer is the result of continual unregulated cell proliferation that leads to the formation of a tumor. This occurs when cells are mutated in a way that affects the signals controlling normal cell behaviour allowing for enhanced cell division to occur. Other signaling pathways that are modified in cancer cells are generally involved in the maintenance of homeostasis, including metabolism, differentiation, survival, and angiogenesis [1]. According to the World Health Organization (WHO), in 2016, cancer was the top cause of disease burden as it was estimated to represent 244.6 million disability-adjusted life years in both men and women. In general, men are more affected by lung and prostate cancer, while women are more affected by breast cancer followed by lung cancer. The WHO estimates that by 2030, cancer malignancies will become the leading cause of mortality worldwide [2].

In cancer, mutagenesis in proto-oncogenes or tumor-suppressor genes typically leads to dysregulated cell signaling and tumor formation. When a proto-oncogene becomes mutated, it is known as an oncogene. In general, conversion of a proto-oncogene to an oncogene is influenced by either a point-mutation, localized reduplication, or chromosomal translocation leading to a gain-of-function mutation. Point mutations may encode proteins that are different to normal proteins. With localized reduplication or chromosomal translocation, there is typically an overabundance in the protein product, thus leading to increased protein expression. These types of mutation are dominant, therefore mutation in only one allele is needed to induce cancer [3].

On the other hand, mutations in tumor-suppressor genes typically result in loss-of-function. Tumor suppressor genes include genes that encode intracellular proteins that regulate or inhibit progression through a specific stage of the cell cycle, receptors for hormones that are secreted to inhibit cell proliferation, check-point control proteins that stop the cell cycle from continuing if there is DNA damage or abnormal chromosomes, pro-apoptotic proteins, and DNA repair enzymes. Mutations in tumor-suppressor genes are typically deletions or point mutations that prevent protein production or produce non-functional proteins. In addition, both alleles must be mutated to promote the formation of a tumor [3].

Once mutagenesis in cancer promoting genes occurs, this can lead to disruption of signaling cascades that are essential in many physiological processes. For example, proteins involved in growth factor receptor tyrosine kinase and nuclear receptor signaling pathways can become activated upon mutagenesis. Disruption of these essential signaling cascades will then disrupt regulated cellular activities and thus promote the formation of tumors [4].

There are several approaches to the treatment of cancer and these treatments vary based on tumor grade, stage, location, and tissue type [5]. In general, traditional cancer treatment options include surgery, chemotherapy, and radiotherapy. Each of these therapies can be used individually or in combination. Although each of these techniques can be effective or even curative in some cases, there are many factors that make cancer treatment difficult. Specifically, it is difficult to target cancer cells exclusively and drug resistance can be a major limitation to traditional cancer treatments. The non-specific nature of cancer symptoms makes diagnosis difficult and tumours often go unnoticed until the cancer has metastasized. Metastasis renders cancer remarkably more difficult to treat, as there will be more sites to target and micro-metastases may be too small to detect [6]. Surgical intervention can be effective but surgery is also extremely invasive and may require the removal of entire organs to achieve disease control without taking into consideration any metastases that may have occurred [7]. Recent advances in cancer treatment have led to the development of more targeted therapies such as immunotherapy which leverages the patient’s own immune system to target cancer cells [8]. However, immunotherapy is not a catchall solution and comes with its own limitations further emphasizing a need to explore novel approaches for both the treatment and prevention of cancer.

### 1.2. Theaflavins as a Potential Anti-Cancer Agent

Many established chemotherapeutics are derived from natural sources. For example, taxanes such as paclitaxel and docetaxel—drugs utilized in the treatment of many cancers—were originally purified from the bark of the Pacific (*Taxus brevifolia*) and the needles of the European (*Taxus baccata)* yew trees, respectively [9]. In recent years, emphasis on healthier eating and lifestyle habits led to the search for natural therapeutic remedies for human diseases, including cancer. Nutraceuticals are a hybrid between nutrition and pharmaceuticals. In general, nutraceuticals are food or food compounds that play a role in maintaining normal physiological functions [10,11]. In cancer, nutraceuticals have the potential to reduce cancer cell growth, inhibit cell proliferation and induce cancer cell apoptosis as many natural and dietary products have been shown to exhibit anti-cancer characteristics [12].

**Figure 1:**
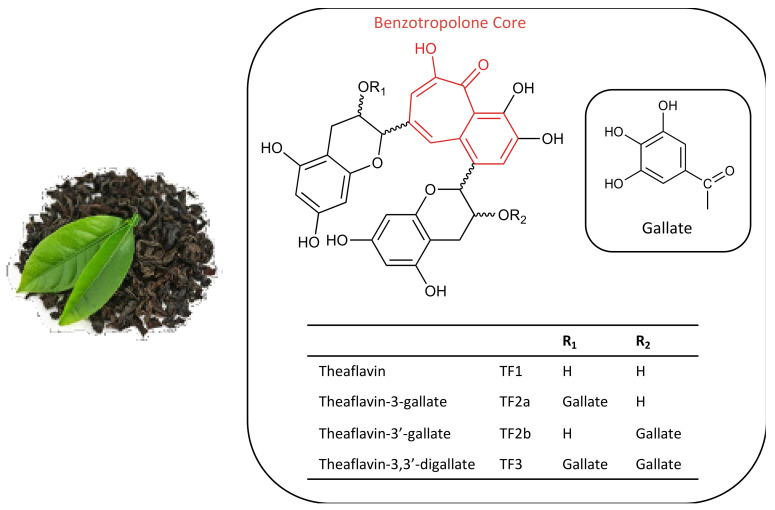
Structure of theaflavins found in black tea. The structure of theaflavin consists of a benzotropolone core (shown in red) formed from the dimerization of a catechin with a gallo-catechin. The theaflavins have two variable R-groups which are substituted with hydroxyl or gallate forming the four main theaflavins: theaflavin (TF1), theaflavin-3-gallate (TF2a), theaflavin-3′-gallate (TF2b), and theaflavin-3,3′-digallate (TF3).

Historically, black tea has been used as a nutraceutical as its components have been shown to possess a variety of health benefits as well as anti-cancer effects in in vitro and in vivo studies [13]. Such effects are largely attributable to the presence of phenolic compounds, including catechins and theaflavins, which account for the highest percentage of biologically active compounds in black tea extracts. Theaflavins are a group of polyphenolic compounds that constitute 2–6% of the dry weight of black tea [14]. They are dimeric catechins formed from the enzyme-catalyzed oxidation and polymerization of tea catechins (flavanols) and there are four main types found in black tea: theaflavin (TF1), theaflavin-3-gallate (TF2a), theaflavin-3′-gallate (TF2b), and theaflavin-3,3′-digallate (TF3) [15]. Structurally, the theaflavins are characterized by a benzotropolone core formed from the dimerization of a catechin and a gallo-catechin (Figure 1) [15].

This review summarizes all available studies on the anti-cancer effects of theaflavins in cancer cell lines and animal models. The studies have been grouped by cancer type and are presented chronologically. The results have been summarized in Tables in each respective section to aid visual comprehension.

## 2. Anti-Cancer Properties of Theaflavins

### 2.1. Theaflavins and Breast Cancer

#### 2.1.1. In Vitro Studies

A study by Way et al. [16] found that treatment of MCF7 breast cancer cells with theaflavins TF1, TF2a/b, and TF3 inhibited aromatase activity (Table 1). Treatment with theaflavins inhibited the DHEA-induced proliferation to the same extent as the aromatase inhibitor 4-OH-A. An anti-proliferative effect was also seen in HER2/neu-transfected MCF7 cells treated with theaflavins which was associated with inhibition of tyrosine phosphorylation of HER2 receptor. Importantly, treatment with theaflavins significantly reduced resistance to tamoxifen, a chemotherapeutic agent commonly used in the treatment of estrogen positive breast cancer [16]. Overall, the results of this study support the role of theaflavins as an aromatase inhibitor suggesting theaflavins may be of use in the treatment of estrogen receptor positive breast cancer.

Lahiry et al. [17] investigated the effects of TF1 treatment on two p53 mutant breast cancer cell lines: MDA-MB-231 and T47D (Table 1). TF1 was found to induce a time-dependent increase in MDA-MB-231cell death. Furthermore, TF1 treatment induced cell cycle arrest (sub-G_0_) [17]. Knockdown of the endogenous mutated p53 in MDA-MB-231 cells had no effect on TF1-induced apoptosis suggesting that TF1 induces apoptosis in a p53-independent manner. Prior treatment of cells with a pan-caspase inhibitor or caspase-9 inhibitor completely abolished the TF1-induced cell death whereas pre-treatment with a caspase-8 inhibitor only partially reduced TF1-induced cell death. TF1 treatment caused a time-dependent increase in activation of caspases-8, -9, and -3, as well as increased protein and mRNA levels of the death receptor Fas. TF1 treatment caused upregulation of phosphorylated c-Jun N-terminal kinase (JNK), whereas siRNA knockdown of JNK1 inhibited the TF1-induced upregulation of Fas [17]. Fas aggregation recruits Fas-associated protein with death domain (FADD) to the plasma membrane and this was also observed following TF1 treatment. Blocking Fas-FADD binding with a Dn-FADD expression vector inhibited TF1-induced cell death, confirming the role of Fas as one of the mediators of TF1-induced apoptosis. TF1 promoted apoptosis in MDA-MB-231 cells as indicated by time-dependent reductions in p-Akt and p-Bad levels. Simultaneous transfection with Dn-caspase-8 and Myr-Akt completely abolished the apoptogenic effects of TF1 proving evidence that these two pathways are responsible for the proapoptotic effect of TF1 in breast cancer cells expressing mutant p53 [17].

Treatment of several breast cancer cell lines (MCF7, MDA-MB-231, and ZR-75-1) with TF1 produced a dose-dependent reduction in cell viability with a greater effect observed in MCF-7 cells which express wildtype p53 and a lesser effect in p53-mutant MDA-MB-231 cells (Table 1) [18]. Treatment of MCF-7 cells with TF1 promoted a time-dependent reduction in viability while inducing apoptosis [18]. TF1 further induced a time-dependent increase in p53 and Bcl-2-associated X protein (Bax) protein expression. Transfection of MCF-7 cells with a dominant-negative p53 gene or p53-siRNA abolished the proapoptotic effect of TF1. When p53 expression was restored in p53-null MDAH041 cells, they became more susceptible to TF1-induced apoptosis [18]. In all cell lines tested, TF1-induced changes in Bax expression correlated with expression of functional p53 suggesting Bax transactivation by p53 due to TF1 treatment. Inhibiting p53-mediated transcriptional activity using pifithrin-α abolished TF1-induced increases in Bax mRNA and protein levels and reduced apoptosis [18]. TF1 treatment caused increased translocation of Bax to the mitochondria with a concomitant decrease in cytosolic levels of Bax while promoting the loss of mitochondrial potential. TF1 also caused increased activation of caspase-9 as well as caspases-6 and -7 [18].

In a study by Adhikari et al., [19] theaflavins inhibited Wt-p53 MCF-7 and ZR-75-1 cell migration in a dose-response manner while upregulating the levels of reactive oxygen species (ROS; Table 1) [19]. Silencing of p53 reversed these effects, indicating the important role of p53 in mediating the anti-cancer effects of theaflavins in breast cancer cells. Theaflavins further enhanced the expression of p53 in MCF-7, ZR-75-1, and MDA-MB-231 cell lines, as well as the levels of proline oxidase while decreasing MnSOD in MCF-7 cells expressing Wt-p53. ROS levels in MDA-MB-231 cells were increased by theaflavins upon transfection with Wt-p53 [19]. It was found that theaflavins could induce p53 phosphorylation at the Ser15 residue whilst this effect was abrogated in the presence of the p38 mitogen activated protein kinase (MAPK) inhibitor SB203580, which also abolished ROS upregulation. Theaflavin treatment inhibited the translocation of NF-kB/p65 to the nucleus of MCF-7 cells, whereas inhibition of p53 expression through siRNA, or N-acetyl cysteine (NAC) treatment reversed this effect. Additionally, the levels of pro-migratory proteins matrix metalloproteinase (MMP)-2 and MMP-9 were downregulated [19].

**Table 1:** Effects of theaflavins in breast cancer.

Cell Line/Animal Model	Treatment	Effects	Reference
Rat ovarian and human ovarian microsomes MCF-7 HER2/neu-transfected MCF-7	5–40 µM TF1, TF2a, TF2b, and TF3	↓Aromatase ↓Cell proliferation ↓Aromatase ↓Tyrosine kinase	[16]
MDA-MB-468	Black Tea Extract 3.1–25 µg/mL 24 or 48 h	↑Apoptosis ↓malondialdehyde-DNA adduct M1dG	[20]
MDA-MB-231 247D	25 µg/mL TF1 4–8 h	↑Cell death ↑sub-G0 cell cycle arrest ↑DNA damage ↑Apoptosis ↑Caspases-8, -9, and -3 ↑Fas ↑p-JNK ↑FADD ↓p-Akt ↓p-Bad	[17]
MCF-7, ZR-75-1, MDA-MB-231	0–37.5 µg/mL theaflavins; 12 h	↓Cell migration ↑ROS ↑p53 ↑Proline oxidase ↓MnSOD ↓NF-kB/p65 nuclear translocation ↓MMP-2 ↓MMP-9	[19]
TAg Mice	0.05% (*w*/*v*) black tea extract in drinking water.	↓Tumour Size ↑Lifespan ↓malondialdehyde-DNA adduct M1dG ↑Cleaved Caspase 3	[20]

Legend: ↑, increase; ↓, decrease.

#### 2.1.2. In Vivo Studies

Kaur et al. [20] demonstrated that oral supplementation (0.05% in diet) of black tea extract (11% TF1, 28% TF2a, 16% TD2b, and 45% TF3) in SV40 T, t antigen transgenic multiple mammary adenocarcinoma (TAg) mice increased mouse survival from 144 days to 154 days paired with a lower number of tumors and decreased tumor volume (Table 1). Histological examination of tumours revealed that the largest tumour in mice that received black tea extract was 40% smaller than the largest tumour in the control group [20]. M_1_dG adducts form as a result of oxidative DNA damage. Treatment of MDA-MB-468 breast cancer cells with black tea extract caused a dose-dependent reduction in M_1_dG adduct levels. Similarly, tumours from mice that received black tea extract had lower levels of M_1_dG adducts compared to tumours from control mice. Staining tumour tissue for proliferating cell nuclear antigen (PCNA) showed that consumption of black tea extract reduced tumour proliferation in the mice by 11% [20]. Overall, these data indicate that theaflavin-rich extracts of black tea can slow and delay the development of cancerous mammary tumours.

### 2.2. Theaflavins and Prostate Cancer

#### 2.2.1. In Vitro Studies

Treatment of prostatic adenocarcinoma LNCaP cells with theaflavins significantly inhibited the expression of the androgen receptor (Table 2) [21]. In addition, significant inhibitory effects on the androgen receptor promotor region were observed [21].

A dose-dependent inhibition of LNCaP cell viability was observed following treatment with TF1, TF2a, TF2b, and TF3 when compared to the untreated control (Table 2) [22]. Of the four theaflavins, TF3 appeared to have the greatest inhibitory effect on cell viability while TF1 had the lowest effect. Testosterone-induced cell growth and androgen receptor expression were reduced upon theaflavins treatment. TF3 induced the greatest inhibition of testosterone-mediated androgen receptor expression while also suppressing testosterone-induced secretion of prostate-specific antigen (PSA). Interestingly, theaflavins reduced the activity of 5α-reductase ex vivo [22]. 5α-reductase catalyzes the synthesis of dihydrotestosterone (DHT) therefore inhibition of DHT synthesis may be a possible mechanism by which theaflavins inhibit PSA secretion.

In a study by Siddiqui et al. [23], a significant inhibition of PI3K and Akt was seen in LNCaP and DU145 prostate cancer cells upon treatment with TF1 (Table 2). Treatment of DU145 cells with TF1 resulted in an upregulation of phosphorylated extracellular signal-regulated kinase (ERK1/2) [23].

Treatment of PC-3 cells with TF1 resulted in decreased cell viability, reduced proliferation and cell cycle arrest at the G2/M cell cycle phase (Table 2) [24]. A decrease in the expression of cyclin B and cdc25C and an increase in p21waf1/cip1 expression was observed, paired with increased levels of cleaved caspase-3 and -9, and Bax and reduced levels of B-cell lymphoma 2 (Bcl-2) proteins, loss of mitochondrial membrane potential and increased DNA fragmentation [24].

Treatment with theaflavins induced apoptosis and loss of mitochondrial membrane potential in PC-3 cells while reducing viability and proliferation (Table 2). In addition, a significant decrease in cytochrome c within the mitochondria, reduced ATP levels and increased caspase-3 activity were observed [25].

Treatment of DU 145 human prostate carcinoma cells with TF1 for 7 days resulted in a dose-dependent reduction in colony formation (Table 2) [26]. 

#### 2.2.2. In Vivo Studies

Male athymic nude mice were implanted with androgen-sensitive human CWR22Rn1 prostate cancer cells and tumors were established [27]. Injection with 1 mg/day of theaflavins resulted in significant inhibition of tumor growth and significant decrease in serum PSA levels (Table 2) [27]. In tumor homogenates, a decrease in the expression of pro-caspase-3 and an increase in active caspase 3 and cleaved PARP were observed, paired with the upregulation of Bax and decrease of Bcl-2 proteins. All these findings indicate enhanced theaflavin-mediated apoptosis. In addition, administration of theaflavins was found to significantly decrease the levels of vascular endothelial growth factor (VEGF) in tumour lysates indicating inhibition of angiogenesis [27]. 

### 2.3. Theaflavins and Lung Cancer

#### 2.3.1. In Vitro Studies

Treatment of human lung cancer cells NCI-H661, NCIF-H441 and NCI-H1299 with a preparation consisting of the four theaflavins (21.4% TF1, 29.9% TF2a, 15.2% TF2b, 27.5% TF3) induced a significant inhibition of growth and reduced cell viability in a dose-dependent manner, with an IC_50_ value of ~20 μg/mL (Table 3). In H661 cells, high doses of theaflavins (100 μM) promoted a significant induction of apoptosis (~77%) [28]. 

Treatment of WI38 VA (SV40 virally transformed lung fibroblasts) with theaflavins caused a dose-dependent inhibition of proliferation (Table 3) [29]. Theaflavins inhibited proliferation to a greater extent in the virally transformed WI38 VA cells compared to non-transformed cells (IC_50_ = 3 µM, 300 µM, respectively). TF2 induced apoptosis in the entire population of WI38 VA cells without affecting non-transformed WI38 cells. Furthermore, DNA fragmentation in the transformed cells could be observed, paired with an inhibition of *COX-*2 expression. These data suggest the potential role of TF2 as promoter of apoptosis in mutated hyperproliferative lung fibroblasts while sparing non-mutated cells.

NCI-H460 human non-small cell lung cancer cells treated with black tea extract theaflavins possessed lower rates of proliferation compared to deionized water-treated cells, as shown by the reduced number of cells labelled with BrdU, a thymidine analog which incorporates within the DNA of S-phase cells (Table 3). Additionally, theaflavin-treated cells had a 10% increase in p53 tumor suppressor expression, paired with the downregulation of the anti-apoptotic protein Bcl-2, and c-Myc [30].

Treatment of SPC-A-1 lung adenocarcinoma cells with TF3 and TF2b+TF3 caused dose-dependent inhibition of viability with IC_50_ of 4.78 and 6.70 µM, respectively. Additionally, TF3 caused significant G_0_/G_1_ phase cell cycle arrest (Table 3) [31].

TF2-induced cytotoxicity in W138VA human SV-40 transformed lung fibroblast cells in a dose-dependent manner paired with DNA fragmentation and cell shrinkage at higher TF2 concentrations (100 μM; Table 3) [32]. Proliferation was inhibited by TF2 with an IC_50_ of 35 µM after 5 days of treatment. Additionally, treatment with TF2 increased p53 and Bax expression, while promoting mitochondrial clustering and formation of vacuoles [32].

SPC-A-1 human lung adenocarcinoma cells treated with TF3 exhibited high rates of apoptosis when compared to a dimethyl sulfoxide (DMSO)-treated control (Table 3) [33]. Comparatively, the activity of caspases-3 and -9 was significantly upregulated in the treated groups when compared to the controls. Pre-treatment with all the 3 MAPK (p38, ERK, JNK) inhibitors and not any combination of two abolished the theaflavin-induced apoptosis indicating that activation of just one of the MAPKs by theaflavins is sufficient to induce apoptosis. [33].

Theaflavins isolated from black tea caused a dose-dependent inhibition of HT 460 human lung cancer cell viability, paired with G2/M phase cell cycle arrest and induced apoptosis (Table 3) [34].

#### 2.3.2. In Vivo Studies

Strain A mice treated with Benzo(a)Pyrene to induce lung tumorigenesis and fed black tea extract containing theaflavins for up to 26 weeks possessed a lower incidence of lung carcinogenesis when compared to the untreated group (Table 3) [35]. Immunohistochemical analysis of tumor tissues revealed that theaflavins significantly inhibited cell proliferation while inducing apoptosis. The apoptotic index of bronchiolar and alveolar tissues was 0.64 in untreated mice and 6.84 in treated mice [35].

### 2.4. Theaflavins and Leukemia

#### 2.4.1. In Vitro Studies

Treatment of human histolytic lymphoma U937 cells with TF1 and TF3 resulted in increased apoptotic body formation and chromatin condensation—clear indicators of enhanced apoptosis (Table 4) [36].

Treatment of U937 cells with TF1, TF2a/b, and TF3 reduced cell proliferation while the same treatment did not affect Jurkat cells (Table 4). Significant morphological changes could be observed in U937 cells, including chromosome condensation and DNA fragmentation, paired with increased PARP cleavage [37].

Treatment of WEHI-3B JSC murine myeloid leukemia cells with the four theaflavin isomers caused dose-dependent inhibition of proliferation (Table 4) [38]. The IC_50_ values ranged from 13.9 to 16.8 µM with the relative anti-proliferative effect of each isomer as follows: TF2b > TF3 > TF2a > TF1. All four TF isomers were also found to exhibit dose-dependent cytotoxic effects. The IC_50_ values ranged from 23.4 to 31 µM with the relative effect the same as listed previously. Reduction of cell survival could only be observed upon treatment with either TF2b or TF3. All TF isomers were demonstrated to induce apoptosis and DNA fragmentation.

A study performed by Tu et al. [39] reported inhibition of acute promyelocytic leukemia (LH-60) cell proliferation by treatment with TF1, TF2b, and TF3 (Table 4) [39].

Darjeeling and Assam black tea extracts and pure TF1 caused dose-dependent inhibition of growth and proliferation of leukemic HL-60 and K-562 cells (Table 4) [40]. Additionally, a dose-dependent reduction in cell viability was demonstrated. Both tea extracts and TF1 showed a dose-dependent induction of DNA fragmentation in both cell lines, paired with increased activation of caspases-3 and -8, higher levels of Bax, and inhibition of Bcl-2 protein levels, indicative of apoptosis [40]. 

Treatment of U937 and K562 human leukemic cells with theaflavins extracted from black tea resulted in the inhibition of cell viability in a dose-dependent manner as a result of cell cycle arrest at the G0/G1 phase (Table 4) [41]. Additionally, theaflavins induced cell apoptosis. At the molecular level, theaflavins enhanced the expression of p21, p19, and p27 while inhibiting cyclin dependent kinase (CDK)2, CDK4, CDK6, cyclin D1 and Hsp90. Theaflavins downregulated the phosphorylation of Akt and Akt’s downstream target GSK-3b, resulting in the reduced expression of nuclear b-catenin, and upregulated the expression of FOXO1 protein. FOXO1 modulation contributed to the anti-proliferative effects of theaflavin, including p27 upregulation, whereas theaflavin-induced attenuation of p-Akt seemed to be partially mediated by its inhibition of Hsp90 [41].

Treatment of human multiple myeloid Arp and Opm1 cells with black tea extract and purified TF1 promoted the inhibition of 20S proteasome activity (Table 4) [42]. Additionally, a dose-dependent inhibition of cell proliferation could be observed. A computational docking study showed it is likely that TF1 binds the β5 subunit of the proteasome [42]. Overall, these data indicate that TF1 is likely responsible for tumour proteasomal inhibition by black tea extract.

#### 2.4.2. In Vivo Studies

WEHI-3B JSC murine myeloid leukemia cells were pretreated with each of the four theaflavin isomers and then injected intraperitoneally into syngeneic BALB/c mice (Table 4) [38]. TF2b inhibited tumor formation to the greatest extent while TF1 was unable to inhibit tumor formation [38].

### 2.5. Theaflavins and Ovarian Cancer

OVCAR-3 ovarian carcinoma cells treated with TF3 exhibited lower rates of proliferation (in a dose-dependent manner) and angiogenesis due to inhibition of VEGF secretion and HIF-1a protein (Table 5) [43]. It is hypothesized that TF3-mediated downregulation of VEGF may occur due to HIF-1 repression. The PI3K/Akt/mTOR pathway, involved in the activation of VEGF though HIF-1a, was inhibited by TF3 as shown by the reduced expression of p-Akt, p-mTOR, p-p70s6k, and p-4E-BP1. In addition, c-Myc, a regulator of HIF-1a, was also suppressed, paired with the reduced cleavage of NOTCH-1, one of its activators [43]. Overexpression of Akt, NOTCH-1 or c-Myc abrogated the TF3-induced inhibition of HIF-1a and VEGF. The expression of MAPK pathway proteins remained unaffected upon treatment [43].

In a study by Gao et al. [44], treatment with either TF1, TF2a, TF2b, or TF3 significantly inhibited the proliferation and induced apoptosis of OVCAR-3 and A2780/CP70 ovarian carcinoma cells (Table 5) [44]. Treatment with theaflavins increased cleaved caspases-3, -7, -8, and -9 levels and the pro-apoptotic Bax protein, as well as FADD and death receptor 5 (DR5), while inhibiting the anti-apoptotic protein B-cell lymphoma extra-large (Bcl-xL) [44]. TF2a, TF2b, and TF3 elicited a greater pro-apoptotic response compared to TF1. Treatment with theaflavins significantly reduced secretion of vascular endothelial growth factor by OVCAR-3 cells and reduced angiogenesis HIF-1a protein expression [44].

A study by Tu et al. [45] demonstrated that TF3 produced higher cytotoxic effects against A2780/CP70 ovarian cancer cellswhen compared to the normal epithelial IOSE-364 cells (Table 5). Additionally, TF3 induced apoptosis in A2780/CP70 cells and increased the activity of caspases 3, -7, while promoting cell cycle arrest at the G2 stage. TF3 upregulated the protein expression of Bax, Bcl-2-associated death promoter (Bad), cleaved caspase-9 and -8, DR5, and FADD while inhibiting Bcl-xL and procaspase-9 and -8, indicating modulation of both intrinsic and extrinsic apoptotic pathways. TF3 further inhibited the protein expression of cyclin B1 and MDM2 and enhanced the expression of p-Akt and the tumor suppressor p53 [45].

The study by Pan et al. [46] investigated the inhibitory role of TF2a and TF2b on the A2780/CP70 ovarian cancer cell lines (Table 5). In a concentration-dependent manner, TF2a and TF2b treatment inhibited cell proliferation, dramatically increased LDH release and increased the percentage of early and late apoptotic cells [46]. Enhanced caspase-3 and -7 activity was observed with TF2a and TF2b treatment. Furthermore, treatment with TF2a and TF2b increased the cell population at the G1 phase and showed a significant decrease in the proportion of cells at the S and G2 phase. More specifically, TF2a was shown to downregulate CDK2 and CDK4 expression and upregulate p21 expression while TF2b was shown to downregulate protein expression of CDK2 and cyclin E1 and upregulate protein expression of p21 [47]. Analysis of the apoptosis results suggest TF2a and TF2b induce G1 phase arrest in A2780/CP70 cells through the downregulation of CDK2 and CDK4 protein expression and CDK2 and cyclin E1 protein expression, respectively. In addition, TF2a and TF2b treatment was shown to upregulate protein expression of p53, p-histone H3A.X (Ser139), p-ATM (Ser1981), p-Chk1 (Ser345), p-Chk2 (Thr68), and p-p53 (Ser15) [47]. TF2a significantly increased ATM protein expression. Treatment with both TF2a and TF2b significantly reduced Akt phosphorylation and ERK1/2 phosphorylation. An increase in p38 phosphorylation and a decrease in JNK expression and phosphorylation was found with TF2a treatment only [47].

In a study by Pan et al. [47], the effects of TF3 in A2780/CP70 and OVCAR3 cells were examined (Table 5). TF3 treatment showed a decrease in the viability of both cell lines in a concentration dependent manner (0–22.5mM). Treatment with 7.5 mM of TF-3 had no significant effect on the protein levels of p-ATM (Ser1981), p-p53(Ser15), p-histone H2A.X (Ser139) indicating the absence of DNA damage. Furthermore, treatment with TF3 decreased the levels of glutathione (GSH) in both ovarian cancer cell lines [47]. Treatment with TF3 also showed no significant effect on the protein levels of the cisplatin transporters MRP2, ATP7A, ATP7B, however, increased the cisplatin transporter CTR1 protein levels in a concentration-dependent manner [47].

Cisplatin-resistant human ovarian cancer cells (A2780/CP70 and OVCAR-3) had reduced proliferation when treated with TF3 (Table 5) [48]. Further investigation showed that TF3 decreased the percentage of A2780/CP70 ALDH+ cells but increased the percentage of OVCAR3 ALDH+ cells in a concentration-dependent manner suggesting a stronger inhibitory effect against A2780/CP70 cancer stem cells than non-cancer stem cells. Treatment with TF3 also increased cleaved caspase-3 and -7 protein levels indicating an increase in apoptosis in both cell lines. Treatment with TF3 significantly decreased β-catenin, LEF-1 (β-catenin target), c-Myc, and cyclin D1 protein levels in both cell lines [48]. 

Pan et al. [49], investigated the effects of TF3 in A2780/CP70 and OVCAR-3 cells and the underlying mechanisms involved (Table 5). TF3 was shown to decrease cell viability and colony formation. An increase in apoptosis in both cell lines upon TF3 treatment was also observed using fluorescence microscopy. TF3 treatment was shown to significantly induce G1 phase arrest and decrease the portion of cells in G2 phase for both cell lines [49]. Furthermore, TF3 treatment significantly decreased protein expression of cyclin D1 and CDK4 but showed no significant effect on cyclin E1 and CDK2 expression [49]. 

Another study by Gao et al. [50] showed that TF3 dose-dependently inhibits the viability of OVCAR-3 cells while upregulating apoptosis. This was confirmed by increased levels of cleaved PARP, Bax, activated caspases-3, -7, -8, -9, and p-Chk2 as well as lower levels of Bcl-xL (Table 5). The expression of death receptors DR5 and Fas were also enhanced. In addition, TF3 caused cell cycle arrest at the G0/G1 stage inhibiting progression to the S phase. This was supported by western blotting indicating lower levels of CDK4, Cyclin D1, phospho-retinoblastoma protein (p-Rb) and Rb in TF3-treated cells whereas p27 expression was enhanced. The pro-apoptotic effects of TF3 were abrogated in the absence of p27 indicating its role in mediating the anti-cancer effects of this compound in ovarian cancer [50].

### 2.6. Theaflavins and Cervical Cancer

HeLa cells treated with TF1 had reduced proliferation and increased apoptosis when compared to the control, untreated group (Table 6) [51]. A decrease in cellular GSH content and an increase in ROS levels were observed with TF1 treatment. Furthermore, an increase in p53 and Bax proteins with a decrease in Bcl-2 and MMP were observed. The researchers also noted an increase in the release of cytochrome c, activated caspase-9, activated caspase-3, and cleaved PARP in HeLa cells treated with TF1 [51]. A decrease in phosphorylated IkBa and, in turn, inactivation and inhibition of nuclear translocation of NF-kB was observed. Cox-2, phosphorylated Akt (Ser473), and cyclin D1 protein levels were decreased in theaflavin-treated HeLa cells [51].

TF2a/b induced cytotoxicity and cell shrinking in HeLa cells in a dose-dependent manner. This was paired with DNA fragmentation at higher TF2 dosages (100 μM; Table 6). Additionally, TF2a/b played a role in the suppression of TPA-induced COX-2 gene expression [32].

In a study by Chakrabarty et al. [52], TF1 treatment induced apoptosis and decreased survival of HeLa cells (Table 6). The levels of p53, cleaved caspase-3, Bax, and cleaved PARP proteins were increased whereas Bcl-2 protein levels were reduced. TF1 inhibited the PI3K/Akt pathway in HeLa cells as shown by lower levels of p85, the regulatory subunit of PI3K, and lower phosphorylated Akt protein levels. A higher population of cells in the G2/M phase was further observed when compared to the control, paired with a drop in the mitochondrial membrane potential [52]. TF1 elicited microtubule depolymerization and increased the levels of soluble tubulin while polymeric tubulin levels were lower. It is suggested that TF1 might bind tubulin at the vinblastine binding site, and that microtubule depolymerization may be an event occurring prior to apoptosis [52].

### 2.7. Theaflavins and Skin Cancer

Treatment of A431 epidermoid carcinoma cells with 1-50 µM TF1, TF2a, TF2b, or TF3 for 30 min each caused dose-dependent inhibition of growth as assessed two days after treatment (Table 7) [53]. Of the four theaflavins, TF3 caused the greatest inhibition of cell growth with an IC_50_ of 18 µM. Pre-treatment with TF3 followed by EGF reduced the EGF-induced activation of epidermal growth factor receptor (EGFR) in a dose-dependent manner with 5 µM sufficient to inhibit receptor kinase activity by 75% and 10 µM causing complete inhibition of EGFR activation. Using a [^125^I]EGF binding assay, it was determined that TF3 pre-treatment and cotreatment for 30 min both reduced EGF binding to its receptor in a dose-dependent manner [53]. These data suggest that TF3 may contribute to the anti-proliferative effect of black tea by reducing the kinase activity of EGFR.

Treatment of human A431 epidermoid carcinoma cells with 20 µM TF3 for 1 h caused internalization of EGFR in EGFR-overexpressing cells as determined with confocal microscopy (Table 7) [54]. An EGF-binding assay showed that the TF3 treatment significantly reduced cell surface expression of EGFR to less than 50% of control. Western blotting of whole cell lysates showed a time-dependent reduction in total levels of EGFR and immunoprecipitation showed a time-dependent increase in levels of ubiquitinated EGFR indicating that TF3 is leading to EGFR being targeted for degradation. TF3 treatment in the presence of a proteasome inhibitor (MG132) abolished the TF3-induced EGFR downregulation [54].

Treatment of A431 and A375 human skin cancer cells with theaflavins extracted from black tea resulted in reduced viability paired with increased apoptotic processes and DNA fragmentation in a dose-dependent manner (Table 7) [55]. Cell cycle analysis revealed a higher number of A375 cells in the G0/G1 stage and fewer in the S stage, indicative of cell cycle arrest. Contrastingly, theaflavins could not induce apoptosis in normal human epidermal NHEK cells under the same conditions [55]. In A375 cells, theaflavins increased Bax translocation to the mitochondria, lowered mitochondrial membrane potential, and increased the accumulation of cytochrome C in the cytoplasm. Additionally, levels of H_2_O_2_ were upregulated, paired with increased activation of caspase-3, caspase-9, and higher levels of cleaved PARP [55].

TF3, but not TF1, TF2a, and TF2b were able to inhibit melanogenesis in B16 mouse melanoma cell lines (Table 7) [56]. No changes in cell viability were observed between treated and untreated groups. TF3 suppressed the mRNA and protein expression of melanocyte-stimulating hormone (αMSH)-induced tyrosinase, an enzyme involved in melanin biosynthesis [56].

Treatment of A375 cells with theaflavins extracted from black tea resulted in reduced cell viability, increased phosphorylated JNK and p38 MAPK, increased caspase-3 activity, and enhanced phosphorylation of MKK3, MKK6, MKK4, and ASK1 (Table 7) [57]. Additionally, theaflavins increased the levels of ROS Treatment with NAC, a potent antioxidant, abolished the effects of theaflavins on cell death, p-JNK, p-p38, MAPK, p-ASK1, and caspase-3 activity. ASK-1, an upstream regulator of p38 and JNK, could play a role in the theaflavin-induced upregulation of p-38 and JNK by ROS [57].

### 2.8. Theaflavins and Colon Cancer 

#### 2.8.1. In Vitro Studies

Treatment of HT-29 human colon cancer cells with TF1, TF2a, TF2b, and TF3 resulted in reduced growth and viability (Table 8) [28].

Lu et al. [29] showed TF1 and TF3 to have a minimal effect on the growth rate of Caco-2 cells whereas 10 and 50 µM TF2 caused a reduction in the growth rate over the course of 8 days (Table 8). Treatment with TF2 suppressed both basal and serum-induced Cox-2 mRNA expression. TF2 was unable to suppress other growth-related genes such as *c-fos, c-myc, TK,* and *PCNA* as well as *BRCA1, BRCA2,* and *Cox-1*. These data indicate a Cox-2–mediated suppression of colon cancer cell growth by TF2.

Expanding on their previous study, Lu et al. [58] used a differential display assay and real-time reverse transcriptase polymerase chain reaction (RT-PCR) to identify *Homo sapiens* regulator of G protein signaling 10 (*RGS10*) as a target which is selectively induced by TF-2 (Table 8). Once *RGS10* was identified as a target gene of TF2 Lu et al. [58] investigated the effects of TF2 on other RGS members. Time-course analysis (0–24 h) showed a TF2-induced expression of RGS10 within 4 h of treatment and increased *RGS16* with a peak effect at 16 h; TF2 had no effect on the *RGS4* gene. Dose-response analysis (0–100 µM TF2) for 16 h showed both *RGS10* and *RGS14* to be dose-dependently induced by TF2; however, higher doses of TF2 were required to induce *RGS14* expression. Additionally, TF3 was found to induce both *RGS10* and *RGS14*, but to a lesser extent than TF2. These data suggest that the G protein-signaling pathway-mediated effects of tea polyphenols may be attributable to their effects on RGS genes.

Treatment of HCT-15 and HT-29 colon cancer cells with 25 µg/mL TF1 for 4–8 h caused a time-dependent induction of cell death as assessed with a trypan blue exclusion assay (Table 8) [17].

TF2a/b reduced the viability of Caco-2 and HT-29 cells in a dose-dependent manner. TF2a/b treatment of TPA-pretreated Caco-2 cells reduced the gene expression of COX-2, TNF-A, ICAM-1, and NFKB (which are upregulated by TPA), as well as iNOS (Table 8). These results indicate the potential role of TF2a/b as an anti-inflammatory agent through suppression of key signaling cascades [32].

TF3 is generally unstable in cell culture conditions so Ding et al. [59] examined the effects of both TF3 and O-TF3 (TF3 which had been preincubated at 37 °C for 3 h) on HCT-116 colon cancer cells (Table 8). Both TF3 and O-TF3 inhibited cell growth, induced apoptosis and cell cycle arrest [59]. In O-TF3-treated cells, the expression of proteins p53, p21, cleaved caspase-3, and cyclin D1 were significantly increased, whereas cyclin E, COX-2, iNOS, and ERK1/2 levels were reduced. O-TF3 had greater anticancer effects compared to TF3 suggesting that TF3 degradation products increase the anti-cancer effects of TF3 [59].

Imran et al. [34] showed theaflavins isolated from black tea caused dose-dependent inhibition of HCT 116 colon cancer cell viability as assessed with an MTT assay (Table 8). Additionally, flow cytometry showed treatment with theaflavins caused significant G2/M phase cell cycle arrest and induced apoptosis.

Treatment of SW620 and SW480 human colon cancer cells with theaflavins (TF1, TF2a, TF3b, and TF3) resulted in increased apoptosis (Table 8) [60]. Treated SW480 cells had G0/G1-cell cycle phase arrest whereas SW620cells exhibited G2/M-phase arrest. Additionally, treatment with theaflavins improved the ROS-scavenging ability of SW480 cells in an AAPH free radical scavenging assay [60]. Theaflavin-treated cells also had increased levels of cyclin D1, cyclin D3, CDK4, and CDK6. Upregulation of p15 and p18 were also observed in SW480 cells.

Theaflavin-containing black tea extract dose-dependently induced cytotoxicity in HCT-116 cells while sparing normal HEK-293 embryonic kidney cells (Table 8) [61]. Additionally, the activity of DNA methyltransferase (DNMT) decreased with increasing concentrations of theaflavins.

#### 2.8.2. In Vivo Studies

In male Swiss albino mice xenografted with EAC colon cancer cells, theaflavin (black tea extract) treatment (intraperitoneal injection or oral gavage) for 10 days slowed growth of EAC tumour xenografts as indicated by decreased tumour volume and weight (Table 8) [61]. Intraperitoneal injection and oral gavage had comparable effects on tumour growth. Additionally, theaflavins increased the percentage of hemoglobin and total RBC while decreasing the number of WBC and multinucleated tumor cells in theaflavin-treated mice. DNMT activity in mice tissue samples was significantly decreased, paired with the reduced expression of DNMT1 [61].

### 2.9. Theaflavins and Liver Cancer

#### 2.9.1. In Vitro Studies

A study performed by Tu et al. [39] reported inhibition of human liver cancer cell BEL-7402 growth by treatment with TF1, TF2b, and TF3 with IC_50_ values of 180, 110, and 160 µM for each theaflavin, respectively (Table 9).

Treatment of human hepatocellular carcinoma cells HCCLM3, Huh-7, and LO2 with theaflavins resulted in reduced viability (Table 9) [62]. Apoptosis was induced in HCCLM3 and Huh-7 cells upon treatment, and this was associated with reduced levels of pro-caspase-3 and pro-PARP, as well as increased levels of cleaved PARP, and caspases-9 and -3 [62]. Treatment of HCCLM3 and Huh-7 cells with theaflavins inhibited migration. Phosphorylated signal transducer and activator of transcription (STAT) 3 levels were reduced along with the STAT3-regulated gene products including Bcl-3, survivin (antiapoptotic proteins), MMP-2 and MMP-9 (invasion related proteins) [62]. IL-6-induced STAT3 phosphorylation was partially reversed upon theaflavin treatment, suggesting STAT3 as an important target of TF. Overall, the data of this study showed suppression of hepatocellular carcinoma growth and metastasis upon theaflavin treatment.

#### 2.9.2. In Vivo Studies

Administration of theaflavins to mice xenografted with HCCLM3 cells resulted in a significant decrease in tumor size (Table 9). In vivo, treatment with theaflavins significantly increased the number of apoptotic cells in tumor tissues [62]. Treatment with theaflavins also showed a decrease in the metastatic ability of HCCLM3 cells.

A study by Sur et al. [63] examined the effects of TF1 on female Swiss albino mice that were exposed to CCl4/N-nitrosodiethylamine (NDEA) carcinogens (Table 9). Mice were administered TF1 orally for either 15 days prior to carcinogen exposure (pre-treatment group), 15 days prior to the carcinogen exposure and continuously for the remainder of the study (continuous treatment group), or daily after the exposure to the carcinogen until the end of the study (post-treatment group) [63]. Histological analysis of the livers of the mice within the pre-treatment group showed restricted carcinogenesis at mild dysplastic stages followed by moderate dysplastic changes between weeks 20–30. Mice within the continuous treatment group showed evidence of chemoprevention with mild dysplastic changes in livers at all time points. Mice within the post-treatment group showed moderate dysplasia at the 10th week followed by restriction at mild dysplastic stages up to week 30. TF1 treatment reduced cell proliferation and increased apoptosis in all treatment groups [63]. AFP and CD44 expression in all treatment groups were significantly reduced as well. TF1 modulated the Wnt pathway by downregulating β-catenin/activated β-catenin expression and up-regulating sFRP1 and APC expression [63]. Analysis of key regulatory genes within the Hh pathway revealed that treatment with TF1 reduced expression of Gli and SMO while upregulating PTCH1 expression. TF1 further decreased cyclin D1, c-Myc, and EGFR expression while increasing E-cadherin expression suggesting that associated Wnt and Hh pathway genes may be important in liver carcinogenesis. [63].

Another study by Sur et al. [64] examined how theaflavin affected tongue and liver carcinogenesis. Female Swiss albino mice were orally administered NDEA, a carcinogen, as well as TF1 for either 15 days prior to the carcinogen exposure (pre-treatment group), 15 days prior to the carcinogen exposure and continuously for the remainder of the study (continuous treatment group), or daily six weeks after carcinogen exposure until the end of the study (post-treatment group; Table 9) [64]. Mice that were pre-treated with theaflavins were shown to maintain body weights until week 20 where they gradually decreased while mice in the continuous and post-treatment groups showed increased body weights after week 10. Histological examination indicated that mice within the pre-treatment and continuous treatment groups exhibited lower tongue and liver carcinogenesis at mild dysplastic stages or moderate dysplastic stages [64]. Mice within the post-treatment group exhibited mild dysplasia in both tongue and liver lesions. Treatment with TF1 reduced the percentage of proliferating cells and increased apoptosis in all treatment groups for both tongue and liver carcinogenesis. Decreased expression of CD44 was also observed in the treatment groups for both tongue and liver lesions [64]. TF1 decreased mRNA expression of β-catenin and increased mRNA expression of sFRP1 and APC in tongue and liver lesions. Additionally, TF1 significantly reduced the mRNA expression of Gli1, SMO, cyclin D1, cMyc, and EGFR, while increasing the mRNA expression of E-cadherin, in both tongue and liver lesions [64]. Overall, the data presented in this study showed that treatment with TF1 can restrict carcinogenesis in the tongue and liver in this mouse model.

### 2.10. Theaflavins and Esophageal Cancer, Stomach Cancer, and Fibrosarcoma

TF3 greatly enhanced apoptosis in Eca-109 human esophageal cancer cells as indicated by nuclear fragmentation and condensation (Table 10) [33]. Pre-treatment with JNK, p38, and ERK inhibitors abolished the pro-apoptotic effects of TF3. Additionally, TF3 treatment upregulated the activity of caspases-3 and -9. These data suggest a potential role of MAPKs in TF3-induced apoptosis [33].

Few studies have investigated the effects of theaflavins on cancers of the stomach. KATO III cells treated with black tea theaflavin extract, TF1, or TF3 exhibited greater levels of apoptosis compared to untreated controls as shown by the presence of apoptotic bodies and DNA fragmentation (Table 10) [65]. In another study, Tu et al. [39] reported inhibition of proliferation of gastric cancer MKN-28 cells by treatment with TF1, TF2b, and TF3 with IC_50_ values of 1.11, 0.22, and 0.25 mM for each theaflavin, respectively (Table 10) [39].

Maeda-Yamamoto et al. [66] investigated the effects of TF1 on cancer metastasis in vitro by treating H1080 human fibrosarcoma cells through a monolayer of human umbilical vein endothelial cells (Table 10). TF1 treatment for 5 h caused dose-dependent inhibition of HT1080 invasion and cell viability with an IC_50_ of 30 µg/mL while TF3 only had a weak inhibitory effect on invasion. TF1 treatment caused a dose-dependent reduction in proMMP-2, active MMP-2, and MMP-9 secretion. Using sodium dodecyl sulfate polyacrylamide gel electrophoresis (SDS-PAGE), MMP-9 and MMP-2 were purified from cell lysates and incubated in activation buffer in the presence of 8–200 µg/mL TF1 for 18 h. This experiment showed TF1 to dose dependently inhibit MMPs. Overall, these data indicate TF1 is able to reduce cancer cell metastasis by inhibiting the activity of MMPs.

## 3. Limitations

Although theaflavins have demonstrated significant anti-cancer potential across various types of cancer in vitro and in vivo, it is important to recognize the limitations to the use of theaflavins in the clinical setting. The studies summarized in this review showed that, generally, cancer cells must be exposed to micromolar concentrations of theaflavins to observe anti-cancer effects. However, micromolar concentrations of theaflavins cannot be achieved through direct ingestion of the compounds themselves. When two volunteers were administered 30 mg of theaflavins orally—the approximate equivalent of 30 cups of black tea—the maximum concentration of theaflavins detected in serum and urine after two hours were both in the femtomolar range [67]. In another study, mice were supplemented with black tea powder (50 mg/g) in their diet for 2 weeks and trace amounts of theaflavins could be detected in tissues of the small intestine, colon, liver, and prostate [68]. While theaflavins do not reach sufficient concentrations in systemic circulation to exhibit an effect, their low intestinal absorption means the intestinal epithelium may be exposed to sufficient concentrations of theaflavins to exhibit anticancer effects [69]. Therefore, as a nutraceutical, black tea polyphenols may be most effective against adenocarcinomas of the colon. Additionally, metabolites from theaflavin digestion by intestinal microflora can reach substantial concentrations in circulation to have beneficial effects, therefore, there is still a benefit to ingesting theaflavins even if the theaflavins themselves are not reaching the systemic circulation at substantial concentrations [69].

While theaflavins may exhibit poor bioavailability, their ability to modulate signaling pathways and clearly promote cancer cell apoptosis should not be ignored or overlooked and more preclinical studies are required. Further investigations should focus on overcoming the barrier of bioavailability to achieve higher concentrations of theaflavins in target tissues. Some success has been observed with the use of encapsulated nanoparticles to improve delivery of other poorly soluble polyphenols such as resveratrol, naringenin, curcumin, and carnosol [70,71,72]. This may offer a potential solution to improve the bioavailability of theaflavins.

## 4. Conclusions

In the studies presented, there is overwhelming evidence that theaflavins decrease cancer cell proliferation, survival, and migration in vitro while promoting cell cycle arrest and apoptosis. In most of the studies examined, these effects were associated with increased levels of cleaved PARP and cleaved caspases-3, -7, -8, and -9 confirming apoptosis. Some studies also reported the anti-proliferative effects of theaflavins to coincide with increased expression of the proapoptotic protein Bax with a concomitant reduction in expression of the antiapoptotic protein Bcl-2 further confirming the proapoptotic potential of theaflavins. Additionally, some studies showed reduced levels of phosphorylated Akt, phosphorylated mTOR and c-Myc, all of which are often upregulated in cancer. Several studies also showed increased expression of the tumor suppressor p53 which tends to be mutated and/or downregulated in cancer. Furthermore, some studies showed reduced expression of MMPs which is likely responsible for the theaflavin-induced reductions in migration and invasiveness. Interestingly, some of the studies showed this proapoptotic effect of theaflavins to be more pronounced in cancerous cells as opposed to comparable non-cancerous cell lines indicating theaflavins could provide a more targeted approach to cancer therapy than conventional chemotherapeutics. 

Overall, the results from the in vitro and in vivo studies summarized in this review indicate both black tea extract and the four theaflavin isomers abundant within this extract to have potent anti-cancer properties. Across many tissues, theaflavins have shown significant anti-proliferative, anti-migration, and pro-apoptotic effects on various types of cancers and in vitro results have shown that theaflavins downregulate major signaling pathways implicated in the hallmarks of cancer. Additionally, in vivo results have shown that these anti-cancer properties of theaflavins hold true within the tumour microenvironment as theaflavin treatment decreases tumorigenesis in several animal models of cancer. Interestingly, theaflavins appear to exert differential effects in different tissues and their anti-proliferative and proapoptotic effects appear to be more potent in cancerous cells compared to healthy cells. Further studies should be conducted to explore different delivery methods such as encapsulated nanoparticles to maximize bioavailability and bio-accessibility. Ultimately, theaflavins and black tea may be suitable as a nutraceutical for the prevention and treatment of cancer.

## Figures and Tables

**Table 2:** Effects of theaflavins in prostate cancer.

Cell Line/Animal Model	Treatment	Effects	Reference
LNCaP	20, 40 µM Theaflavins 24 h	↓Androgen receptor	[21]
LNCaP	TF1, TF2a, TF2b, and TF3 10–40 µM 1–4 days	↓Cell viability ↓Androgen receptor ↓PSA	[22]
LNCaP, DU145	10, 25, 50 µg/mL TF1; 12 24 h	↓PI3K ↓p-Akt ↑p-ERK1/2	[23]
PC-3	40–80 µg/mL TF1 24–48 h	↓Cell viability ↑G2/M cell phase ↓G0/G1 cell phase ↓Cyclin B ↓cdc25C ↑p21waf1/cip1 ↑Cleaved caspase-3, -9 ↑Bax ↓Bcl-9 ↓Mitochondrial membrane potential	[24]
PC-3	0.4–2.0 µg/mL theaflavins; 24–36 h	↓Cell viability ↓Cell proliferation ↑Apoptosis ↓Mitochondrial membrane potential ↓Cytochrome c ↓ATP ↑Caspase-3	[25]
DU 145	TF1 100, 200 µg/mL 7 days	↓Colony count	[26]
CWR22Rn1 cells in Athymic nude mice	1 mg/day/mice	↓Tumor growth ↓[PSA] ↓Pro-caspase 3 ↑Caspase-3 ↑Cleaved PARP ↑Bax ↓Bcl-2 ↓VEGF	[27]

Legend: ↑, increase; ↓, decrease.

**Table 3:** Effects of theaflavins in lung cancer.

Cell line/Animal Model	Treatment	Effects	Reference
NCI-H661, NCI-H441, NCIH1299	0–100 μM TF1, TF2a, TF2b, and TF3 24–48h	↓ Cell viability ↓ Cell growth ↑ Apoptosis	[28]
WI38 VA	TF2 1–100 µM 18 h–6 days	↓ Proliferation ↓ Growth Rate ↑ Apoptosis ↑ DNA Fragmentation ↓Cox-2 expression	[29]
NCI-H460	100 µM theaflavins 24–72 h	↓ Proliferation ↑ Apoptosis ↑ p53 gene and protein ↓ Bcl-2 gene and protein ↓ c-Myc gene and protein	[30]
SPC-A-1	TF2b+TF3 TF3 48h	↓ Cell viability ↑ G0/G1 Cell cycle arrest	[31]
WI38VA	0–50 μM theaflavin-2; 5 days to assess cell viability/cytotoxicity 0–400 μM theaflavin-2; 3 h–48 h to assess DNA fragmentation, gene expression and mitochondrial morphology	↑ Cytotoxicity ↓ Cell viability ↑ DNA fragmentation	[32]
SPC-A-1	50 µM TF3 24 h	↑ Apoptosis ↑ Caspase-3 and -9 activity	[33]
HT460	TF extract	↓ Viability ↑ G2/M cell cycle arrest ↑Apoptosis	[34]
Strain A mice injected with benzo(a)pyrene to induce lung carcinogenesis	0.02 mg black tea extract/day for 8, 17, and 26 weeks.	↓ Lung carcinogenesis ↓ Proliferation ↑ Apoptosis	[35]

Legend: ↑, increase; ↓, decrease.

**Table 4:** Effects of theaflavins in leukemic cancers.

Cell Line/Animal Model	Treatment	Effects	Reference
U937	100 µM TF1 and TF3 16 h	↑Apoptosis	[36]
U937	0–100 µM TF1, TF2a, TF2b, and TF3 12 h	↓Cell growth ↑PARP cleavage	[37]
WEHI-3B JCS	TF1, TF2a, TF2b, and TF3 10–20 µM 48 h	↓Proliferation ↑Cytotoxicity ↓Clonogenic Survival ↑DNA fragmentation ↑Apoptosis	[38]
LH-60	3.9, 16, 63, 250, 100 µg/mL TF1 TF2b, and TF3	↓Proliferation	[39]
HL-60 K-562	1–1000 µg/mL TF1 or black tea extract 24 h	↓Growth ↓Proliferation ↓Cell viability ↑DNA Fragmentation	[40]
U937, K562	0–100 μg/mL theaflavins; 24 h ½ IC50: 22.3 μg/mL for U937 and 25.1 μg/mL for K562 cells; 24 h IC50: 44.6 μg/mL for U937 and 50.2 μg/mL for K562 cells; 24 h	↓Cell viability ↑Apoptosis ↑Cell cycle arrest at the G0 ⁄G1 phase ↑p21 ↑p19 ↑p27 ↓CDK2 ↓CDK4 ↓CDK6 ↓Cyclin D1 ↓Hsp90 ↓P-Akt ↓Gsk-3b ↓Nuclear b-catenin ↑FOXO1	[41]
Arp Opm1	T5550 Black Tea Extract 10–20 µg/mL 24 h	↓20S proteasome activity ↓Proliferation	[42]
Syngeneic BALB/c mice (WEHI-3B JCS xenograft)	Pre-treatment with TF1, TF2a, TF2b, and TF3 10–20 µM 48 h	↓Tumorgenicity	[38]

Legend: ↑, increase; ↓, decrease.

**Table 5:** Effects of theaflavins in ovarian cancer.

Cell Line/Animal Model	Treatment	Effects		Reference
OVCAR-3	0–25 µM TF3 24 h	↓ Cell viability ↓ Angiogenesis ↓ VEGF ↓ HIF-1a ↓ p-Akt	↓ p-mTOR, ↓ p-p70s6k ↓ p-4E-BP1 ↓ c-Myc ↓ NOTCH-1 cleavage	[43]
OVCAR-3, A2780/CP70	0–40 µM TF1, TF2a, TF2b, and TF3 24 h	↓ Proliferation ↑ Apoptosis ↓ Angiogenesis ↓ VEGF secretion ↓ HIF-1a ↑ Caspases-3, -7, -8, and -9 activity	↑ Bax ↑ FADD ↑ DR5 ↓ Bcl-xL ↓ HIF-1a	[44]
A2780/CP70	TF3	↑ Cytotoxicity ↓ Cell viability ↑ Apoptosis ↑ Caspases-3, -7 activity ↑ Cell cycle arrest at the G2 phase ↑ Bax ↑ Bad ↑ Cleaved caspase-9 ↑ Cleaved caspase-8	↑ DR5 ↑ FADD ↓ Bcl-xL ↓ Procaspase-8 ↓ Procaspase-9 ↓ Cyclin B1 ↓ MDM2 ↑ p-Akt ↑ p-53	[45]
A2780/CP70	0–40 µM TF-2a 24 h	↓ Proliferation ↓ Cell viability ↑ LDH ↑ Early phase apoptosis ↑ Late phase apoptosis ↑ Caspase -3, -7 activity ↓ Pro-caspase-3, -7 ↑ Cleaved caspase-3, -7 ↑ Full length PARP ↑ Cleaved PARP ↑ G1 phase ↓ S phase ↓ G2 phase ↓ CDK2	↓ CDK4 ↑ p21 ↑ p53 ↑ p-histone H2A.X ↑ p-ATM ↑ ATM ↑ p-Chk1 (Ser345) ↑ p-Chk2 (Thr68) ↑ p-p53 (Ser15) ↓ Akt ↑ p-p38 ↓ ERK1/2 ↓ JNK2	[46]
A2780/CP70, OVCAR-3	2.5–22.5 µM TF3 24 h	↓ Cell viability ↓ GSH↑ CTR1		[47]
A2780/CP70, OVCAR-3	5–20 µM TF3 24 h	↓ Cell viability ↓ Cell colonies ↓ Proliferation ↑ %ALDH+ cells ↑ Apoptosis	↑ Caspase-3, -7 ↓ β-catenin ↓LEF-1 ↓c-Myc ↓Cyclin D1	[48]
A2780/CP70, OVCAR-3	5–50 µM TF3 24 h 7.0 µM TF3 24 h	↓ Cell viability ↓ Cell colonies ↑ Apoptosis	↑ G1 phase arrest ↓ G2 phase cells ↓ Cyclin D1↓ CDK4	[49]
OVCAR-3	0–30 μM TF3 24 h	↑ Apoptosis ↓ Cell viability ↑ Cleaved PARP ↑ Bax ↑ Cleaved caspases-3, -7, -8, and -9 ↑ p-Chk2 ↓ Bcl-xL ↑ DR5	↑ Fas ↑ Cell cycle arrest at the G0/G1 phase ↓ CDK4 ↓ Cyclin D1 ↓ p-Rb ↓ Rb ↑ p27	[50]

Legend: ↑, increase; ↓, decrease.

**Table 6:** Effects of theaflavins in cervical cancer.

Cell Line/Animal Model	Treatment	Effects	Reference
HeLa	5–50 µg/mL TF1 24 h	↓Cell proliferation ↑Apoptosis ↑Sub-diploid DNA ↑Early phase apoptosis ↑Late phase apoptosis ↓GSH ↑ROS ↓MMP ↑p53 ↑Bax ↓Bcl-2 ↑Cytochrome c ↑Activated caspase-9 ↑Activated caspase-3 ↑Cleaved PARP ↓IkBa ↓NF-kB ↓Cox-2 ↓p-Akt ↓Cyclin D1	[51]
HeLa	0–400 μM TF2a/b 3 h–5 days	↑Cytotoxicity ↓Cell size ↑DNA fragmentation ↓COX-2 mRNA	[32]
HeLa	10–500 μg/mL TF1 0–48 h	↓Cell survival ↑Apoptosis ↑p53 ↑Cleaved caspase-3 ↑Bax ↑Cleaved PARP ↓Bcl-2 ↓p85 subunit of PI3K ↓p-Akt ↑Cell cycle arrest at the G2/M stage ↓Mitochondrial membrane potential ↑microtubule depolymerization ↑Soluble tubulin ↓Polymeric tubulin	[52]

Legend: ↑, increase; ↓, decrease.

**Table 7:** Effects of Theaflavins on Skin Cancer.

Cell Line/Animal Model	Treatment	Effects	Reference
A431	TF3 0.1–50 µM 10–30 min	↓Growth ↓EGFR kinase activity ↓EGF Binding	[53]
A431	TF3 20 µM 2 min–1 h	↑EGFR internalization ↓EGFR expression ↑EGFR ubiquitination ↓EGF-induced EGFR activation	[54]
A431 A375	0–100 μg/mL Theaflavins (black tea extract) 0–48 h	↓Cell viability ↑Apoptosis ↑DNA fragmentation ↑Cell cycle arrest at the G0/G1 phase ↑Bax translocation to mitochondria ↓Mitochondrial membrane potential ↑Cytoplasmic cytochrome C ↑H_2_O_2_ ↑Cleaved caspase-3 ↑Cleaved caspase-9 ↑Cleaved PARP	[55]
Mouse B16 melanoma 4A5 cells	0–20 μM TF1, TF2a, TF2b, TF3 0–72 h	↓Melanogenesis ↓Tyrosinase mRNA and protein	[56]
A375	0–100 μg/mL Theaflavins (black tea extract) 0–48 h	↓Cell viability ↑p-JNK ↑p-p38 MAPK↑Caspase 3 activity ↑p-MKK3 ↑p-MKK4 ↑p-MKK6 ↑p- ASK1 ↑ROS	[57]

Legend: ↑, increase; ↓, decrease.

**Table 8:** Effects of theaflavins in colon cancer.

Cell Line/Animal Model	Treatment	Effects	Reference
HT-29	0–100 μM; 48 h	↓Cell viability ↓Cell growth	[28]
Caco-2	10–100 µM TF2 4 h–8 days	↓Growth rate ↓Cox-2 expression	[29]
Caco-2	TF2 50 µM 12 h	↑RGS10 expression ↑RGS14 expression	[58]
HCT-15 HT-29	25 µg/mL TF1 4–8 h	↑Cell death	[17]
Caco-2 HT-29	0–400 μM TF2a/b 3 h–5 days	↓Cell viability ↓COX-2 mRNA ↓TNF-A mRNA ↓ICAM-1 mRNA ↓NFKB mRNA ↓iNOS mRNA	[32]
HCT-116	0–30 TF3 3–48 h	↓Cell viability ↓Cell proliferation ↑Apoptosis ↑Cell cycle arrest at the G2 phase ↑p53 ↑p21 ↑Cleaved caspase-3 ↑Cyclin D1 ↓Cyclin E ↓COX-2 ↓iNOS, ↓ERK1/2	[59]
HCT 116	Theaflavin extract	↓viability ↑G2/M cell cycle arrest ↑apoptosis	[34]
SW620 SW480 NCM460	45 µM TF1, TF2a, TF3b, and TF3 12 h	↓Cell growth ↓Cell size ↑Apoptotic bodies ↑G0/G1 phase arrest ↑G2/M phase arrest ↓ABTS•+ ↓DPPH •+ ↓AAPH ↑Cyclin D3 ↑Cyclin D1 ↑CDK4 ↑CDK6 ↑p15 ↑p18	[60]
HCT-116	Theaflavins (black tea extract) 25–150 μg/mL 48h	↓Cell viability ↓DNMT activity ↓DNMT1	[61]
Male Swiss albino mice xenografted with EAC cells.	Theaflavin intraperitoneal injection, 0.02 mg/kg body weight/day for 10 days Theaflavin oral gavage at a dose of 10 mg/kg body weight for 10 days	↓Tumor growth/mass ↑Haemoglobin ↑RBC count ↓WBC count ↓Multinucleated tumor cells ↓DNMT activity ↓DNMT1	[61]

Legend: ↑, increase; ↓, decrease.

**Table 9:** Effects of theaflavins in liver cancer.

Cell Line/Animal Model	Treatment	Effects	Reference
Human liver BEL-704	3.9–250 µg/mL TF1, TF2a, TF2b, and TF3	↓ Cell growth	[39]
HCCLM3, Huh-7, and LO2	0–80 µg/mL theaflavins 24–28 h	↓ Cell viability ↑ Apoptosis ↑ Cleaved-caspase 9 ↑Cleaved-caspase 3 ↑Cleaved-PARP ↓Pro-caspase-3 ↓Pro-PARP ↓p-STAT3 ↓STAT ↓Bcl-3 ↓Survivin ↓MMP-2 ↓MMP-9	[62]
Male BALB/c athymic mice (CWR22Rn1 cells)	10 mg/kg/d theaflavins peritoneal injection daily for 5 weeks	↓Tumor size ↑ Apoptosis ↓Metastasis	[62]
Female Swiss albino Mice (intraperitoneal injection of CCl_4_/NDEA)	10 µg/kg body weight (oral) TF1 Pre-treatment group: daily for 15 days prior to carcinogenesis Continuous treatment group: daily for 15 days prior and throughout study (10–30 weeks) Post-treatment group: daily following carcinogenesis until end of study (10–30 weeks)	↓ Cell proliferation ↑ Apoptosis ↓ AFP ↓ CD44 ↓ β-catenin ↑ sFRP1 ↑ APC ↓ Gli↓ SMO ↑ PTCH1 ↓ Cyclin D1 ↓ c-Myc ↓ EGFR ↑ E-cadherin	[63]
Female Swiss albino mice (oral NDEA)	10 µg/kg body weight (oral) TF1 Pre-treatment group: daily for 15 days prior to carcinogenesis Continuous treatment group: daily for 15 days prior and throughout study (10–30 weeks) Post-treatment group: daily, 6 weeks after carcinogenesis until end of study (10–30 weeks)	↓ Cell proliferation ↑ Apoptosis ↓ mRNA β-catenin ↑ mRNA sFRP1 ↑ mRNA APC ↓ mRNA Gli ↓ mRNA SMO ↓ mRNA cyclin D1 ↓ mRNA c-Myc ↓ mRNA EGFR ↑ mRNA E-cadherin	[64]

Legend: ↑, increase; ↓, decrease.

**Table 10:** Effects of theaflavins in esophageal cancer, stomach cancer and fibrosarcoma.

Cell Line/Animal Model	Treatment	Effects	Reference
Eca-109	50 µM TF3 24 h	↑Apoptosis ↑Caspase-3 and -9 activity	[33]
KATO III	0–1 mg/mL theaflavin extract; 1–3 days 0–1.5 mM TF3; 1–3 days 0–2 mM TF1; 1–3 days	↑Apoptosis ↑Apoptotic bodies ↑DNA fragmentation	[65]
MKN-28	3.9, 16, 63, 250, 100 µg/mL TF1 TF2b, and TF3	↓Proliferation	[39]
H1080	TF1 0.8–100 µg/mL 5–18 h	↓Invasion ↓Cell Viability ↓MMP-9 and -2 activity	[66]

Legend: ↑, increase; ↓, decrease.

## Sample Availability

Samples of the compounds are available from the authors.
